# Epigenomics of Pancreatic Cancer: A Critical Role for Epigenome-Wide Studies

**DOI:** 10.3390/epigenomes3010005

**Published:** 2019-01-19

**Authors:** Rahul R. Singh, Katie M. Reindl, Rick J. Jansen

**Affiliations:** 1 Department of Biological Sciences, North Dakota State University, Fargo, ND 58102, USA; 2 Department of Public Health, North Dakota State University, Fargo, ND 58102, USA; 3 Biostatistics Core Facility, North Dakota State University, Fargo, ND 58102, USA; 4 Center for Immunization Research and Education, North Dakota State University, Fargo, ND 58102, USA; 5 Genomics and Bioinformatics Program, North Dakota State University, Fargo, ND 58102, USA

**Keywords:** epigenetics, epigenomes, pancreatic cancer, methylation, histones, RNA, DNA, expression

## Abstract

Several challenges present themselves when discussing current approaches to the prevention or treatment of pancreatic cancer. Up to 45% of the risk of pancreatic cancer is attributed to unknown causes, making effective prevention programs difficult to design. The most common type of pancreatic cancer, pancreatic ductal adenocarcinoma (PDAC), is generally diagnosed at a late stage, leading to a poor prognosis and 5-year survival estimate. PDAC tumors are heterogeneous, leading to many identified cell subtypes within one patient’s primary tumor. This explains why there is a high frequency of tumors that are resistant to standard treatments, leading to high relapse rates. This review will discuss how epigenetic technologies and epigenome-wide association studies have been used to address some of these challenges and the future promises these approaches hold.

## 1. Introduction

Consistently across sources, the top identified risk factors for pancreatic cancer include: Smoking, obesity, age, recent onset diabetes mellitus, recent pancreatitis, hereditary pancreatitis and hereditary pancreatic cancer. These risk factors are only present in about 50–60% of pancreatic cancer cases, with known genetic alterations only accounting for about 5–10% [1,2,3,4]. Investigation into the role of dysregulated epigenetic processes could help explain some of the unknown causes.

Globally in 2018 for pancreatic cancer, the estimated number of new cases was over 1,000,000, with 65,000 deaths [5]. Pancreatic ductal adenocarcinoma (PDAC) is the most common form of pancreatic cancer, representing about 90% of pancreatic cancer cases. PDAC is generally diagnosed at a late stage, leading to an extremely poor 5-year survival rate of around 7% [5]. PDAC tumors are heterogeneous and frequently are resistant to standard treatments [6]. Currently, no effective biomarkers have been identified for use in clinical diagnosis or prognosis.

PDAC is characterized by well-defined genetic modifications; however, epigenetic alterations have recently been recognized as important contributors to PDAC development and progression [7], as well as potential therapeutic targets [8]. Epigenetic changes are heritable modifications that are made to the DNA chemistry or chromatin structures, influencing gene expression without altering the DNA sequence [9]. Epigenetic alterations to oncogenes and tumor suppressor genes affect tumor progression and are associated with PDAC patient survival post-diagnosis [10]. The principle epigenetic mechanisms that influence tumor-associated gene expression are: DNA methylation [11]; histone modification, including histone acetylation, deacetylation and methylation [12]; and microRNAs [13].

Epigenetics refers to processes that take action on the DNA, rather than modifying the DNA sequence (i.e., genetic alterations). Epigenomics only refers to the analysis of these epigenetic processes across multiple genes or the entire genome (i.e., whole genome-wide). Several schematics exist to demonstrate the interrelationship between genomic, epigenomic and signaling pathway alterations in PDAC [14,15,16,17]. In this review, we will describe the different epigenetic processes and then move to focus on epigenome-wide technologies and epigenome-wide association studies (EWAS) that have investigated epigenetic markers associated with either the diagnosis or prognosis of pancreatic cancer.

## 2. Epigenetic Processes

In this section, several types of epigenetic processes are discussed, along with current laboratory methods used to detect markers of each type of process. The processes that are discussed include nucleosome remodeling complexes and nuclear architecture, histones, transcription factors, methylation and non-coding RNA.

### 2.1. Nucleosome Remodeling Complexes and Nuclear Architecture

Nuclear DNA exists in a very compact configuration because of its interaction with an array of proteins, including histones. During the process of transcription and replication, chromatin is relaxed, remodeled and recovered in a cascade of events concisely referred to as chromatin remodeling (CR). CR can be initiated by various types of histones or through an ATP-dependent mobilization of nucleosomes; the latter is termed as nucleosome remodeling (NR). NR complexes are categorized into four families: SWI/SNF, ISWI, CHD and INO80, each harboring a family-specific ATPase, BRM or BRG1 (SWI/SNF), SNF2H (ISWI), CHD3 (CHD) and INO80 (INO80). Increasing evidence now suggests that NR has fundamental roles in transcriptional regulation and DNA-damage repair [18]. The subunits of NR complexes are known to be mutated or replaced with other complexes in more than 20% of human cancers [19,20].

The basic biological properties of nucleosomes and NR are investigated using atomic force microscopy (AFM) [21]. Additionally, DNA-histone binding strength, nucleosome stability and nucleosome locations can also be analyzed using AFM [22,23]. More recent methods and technique enhancements have been developed, including 3D modeling [24] and visualization in a buffer without fixation [25].

### 2.2. DNA-Protein Interaction

#### 2.2.1. Histone Modifications

Histone modifications, including acetylation and methylation, are another major epigenetic mechanism responsible for regulating gene expression. The acetylation of lysine residues within histone proteins prevents the positively charged lysine from interacting with the negatively charged DNA, resulting in a more open chromatin structure that promotes gene expression. Conversely, deacetylation has the opposite effect and results in a more closed chromatin structure that suppresses gene expression. Histone deacetylases (HDACs) and histone acetyltransferases (HATs) are the key enzymes associated with the removal and addition of acetyl groups to lysine residues in histone proteins [26,27]. Increased expression of various HDACs is observed in PDAC and associated with reduced tumor suppressor gene expression and enhanced cancer cell proliferation [28,29]. Enhanced HAT activity can activate oncogenes that drive tumor growth [30]. The methylation of arginine and lysine residues in histone proteins constitutes another form of histone modification. Several methyltransferases and demethylases are known to be overexpressed, mutated, deleted and dysregulated in various human cancers; however, their impact on cancer-related mortality is not clearly demonstrated [31]. 

The aberrant expression levels of mucinous glycoproteins, such as MUC17, has earlier been correlated with PDAC pathogenicity and progression [32]. Epigenetic modifications, such as the dimethylation of H3-K9 in histone proteins, reduce the expression of MUC17, while acetylation at the same sites was shown to elevate the levels of MUC17 [33]. Additionally, a higher activity of histone alterations, like H2AK119 monoubiquitination (H2AK119Ub1) and H3K27 trimethylation (H3K27Me3), which are controlled by Polycomb group (PcG) complexes, is associated with poor prognoses and shorter survival times in PDAC patients [34].

Techniques to evaluate histone modifications frequently involve targeted antibodies [35]. Although this approach is sensitive and specific, prior knowledge of these modifications is required. Affinity pull-down assays use different baits such as peptides, nucleosomes and chromatin to identify histone modifications [36,37]. Chromatin immunoprecipitation and the subsequent sequencing of the DNA fragments, referred to as chromatin immunoprecipitation sequencing, or ChIP-Seq, can analyze genomic regions that are enriched in a particular histone modification. A critical challenge exists in the development of sophisticated bioinformatics tools to integrate genomics and proteomics data and provide new visualization tools for a comprehensive representation of chromatin organization [38].

#### 2.2.2. Transcription Factors

Transcriptional factors (TFs) have the functional role of directing and attaching transcriptional machinery to specific segments of the DNA within the cell [39]. Several TFs have been hypothesized to influence pancreatic cancer differentiation and development including: Pancreas/duodenum homeobox protein 1 (PDX1), pancreas transcription factor 1 subunit alpha (PTF1A), nuclear receptor subfamily 5 group A member 2 (NR5A2), hepatocyte nuclear factor 1-alpha (HNF1A) and hepatocyte nuclear factor 1-beta (HNF1B) [40]. Certain sequence-specific TFs, such as KLF11 and KLF14, have been observed to primarily regulate metabolic gene networks [41,42,43] and genes important in PDAC (e.g., KRAS) have been observed to either influence, or be influenced by, the functions of TFs [40,44]. More specifically, acinar cells lacking PTF1A are more frequently transformed by KRAS [45,46], while NR5A2 prevents KRAS induced neoplasia through the maintenance of acinar cell plasticity [47], and PDAC metastasis has been observed to increase with the activation of KRAS and the expression of c-Myc TFs [48]. TFs have also been identified to influence PDAC survival or drug resistance through their functional role in directing transcriptional machinery, thus providing potential individualized therapeutic targets [49].

#### 2.2.3. Next Generation Technologies

ChIP-Seq can be used to survey interactions between proteins, DNA and RNA. ChIP-Seq using next generation sequencing (NGS) enables researchers to identify the binding sites of multiple protein targets, including transcription factors and histones, across the entire genome. Comprehensive tools such as Spatial Clustering for Identification of ChIP-Enriched Regions (SICER) [50] and Model-based Analysis for ChIP-seq (MACS) [51] have been used to identify differential epigenetic markers in PDAC patients using ChIP-Seq data. Several challenges have been identified for the analysis of ChIP-Seq data, including understanding alterations based on different population genetic architectures, understanding existence of modifiers or buffering variants and understanding missing heritability [52]. It will be essential to overcome these challenges to deepen our understanding of the importance of specific DNA-protein interactions and to identify the functionally relevant DNA and protein modifications which create microenvironmental conditions favorable for PDAC development and growth [53].

### 2.3. DNA Methylation

DNA methylation involves the addition of a methyl group to the 5-carbon position of cytosine within the DNA sequence and most frequently occurs in CpG sites where a cytosine (C) is positioned next to a guanidine (G) nucleotide. DNA methyltransferases (DNMTs) initiate and maintain the methylation state of the CpG sequences, which are commonly present in the promoter regions of genes. Promoter hypermethylation is primarily associated with gene silencing, as methylation interferes with the transcription factor binding to the promoter, resulting in the suppression of gene expression [54]. Conversely, promoter hypomethylation is associated with an increased expression of the corresponding gene product. Cancer-associated genes, such as tumor suppressor genes, frequently display promoter hypermethylation which reduces their expression and results in unchecked tumor cell proliferation [55,56]. Recently, it was reported that the overexpression of DNMT1 in PDAC was responsible for silencing key tumor-suppressor genes; namely p16, preproenkephalin and Ras association domain family member 1 [57]. Critical cytokines such as transforming growth factor beta 1 (TGF-β1) are known to drive epithelial-to-mesenchymal (EMT) transition in pancreatic cancer cells [58,59]. The histone methyl transferase enzyme EZH2 was recognized to regulate EMT, mediated via TGF-β1 signaling [60]. Additionally, the aberrant and widespread loss of DNA methylation of several transcription factors such as TFF1, TFF2 and E2F5 has been identified in PDAC tissues [61].

Techniques to evaluate DNA methylation have evolved since the 1970’s, when the first approaches were developed [62]. These early approaches included methylation-specific restriction enzyme digestion and affinity purification of methylated DNA. Most of the techniques currently used rely on bisulfite treatment and a subsequent PCR or sequencing analysis. The bisulfite method converts cytosines to thymidines and can thereby distinguish methylation levels at a single-base-pair resolution [63]. Next generation Illumina and Roche sequencing platforms have been commonly used to sequence whole genomes after bisulfite treatment [64]. Methylated DNA fragments can also be isolated using immunoprecipitation and are further used for hybridization with microarrays [65]. Several reviews have recently summarized DNA methylation technologies [66] and the decision of which technique to use can be made based on the information required, number and type of samples, and cost [67,68]. 

Recently, several studies (last two in preprint stage) have observed increases in both widespread and targeted gene-specific 5-hydroxymethylation in PDAC. 5-hydroxymethylcytosine (5-hmC) is a residue that is generated from the well-studied cancer associated 5-methylcytosine (5-mC). The first study used a mass spectrometry method to compare 11 patient derived xenografts (PDX), 11 PDAC cell lines, and two control pancreas cell lines. Their results suggested an increase in 5-hmC in the enhancer region of key PDAC oncogenic genes (e.g., MYC, KRAS, VEGFA, and BRD4) [69]. The second study used the high-throughput Illumina EPIC array to describe differences in 5-hmC distributions in 17 pairs of patient pancreas tumor/adjacent tissues and found enrichment in genes found in pathways relevant to cancer [70]. The last study used the Illumina NextSeq550 instrument with version 2 reagent chemistry to compare 5-hmC density differences in cell-free DNA from 51 PDAC patients and 41 non-cancer patients. They identified (and validated in two independent datasets) a set of four pancreas related genes and seven cancer related genes which differentiate between PDAC and normal patients, with a reasonable Area Under the Curve (AUC) between 0.74–0.97 [71]. These results in total suggest an important regulatory role for 5-hmC in the development and growth of PDAC.

### 2.4. Non-Coding RNA

Protein-coding genes constitute only 2% of the human genome, while a major proportion is transcribed into non-coding RNAs (ncRNAs). NcRNA refers to a broad category of RNA molecules that includes circular-RNA (circRNA), piwi-RNA (piRNA), micro-RNA (miRNA), small-interfering RNA (siRNA), enhancer-RNA (eRNA) and long non-coding RNA (lncRNA). Substantial breakthroughs have elucidated the functions of ncRNAs and have supplied plentiful evidence suggesting the roles of ncRNAs in all fundamental cellular processes [72,73,74]. For the past two decades, many studies have provided convincing data suggesting the roles of ncRNAs in tumor development, progression [75], metastasis [76] and in drug-resistance [77]. Small RNA sequencing is commonly used to investigate the abundance and sequence of small RNAs and shares the same basic principle and sample preparation techniques as mRNA sequencing. Simple and cost-effective solutions, such as TruSeq Small RNA Library Prep methods, are now available to generate small RNA libraries from total RNA. With the development of deep sequencing platforms by Illumina and Roche, it is now possible to identify and quantify small RNAs with unprecedented sensitivity and resolution. Using the aforementioned small RNA sequencing platforms, the roles of various miRNAs have now been validated in PDAC cell invasion [78], migration [79], EMT [80] and resistance to chemotherapy [81]. Whole genome high-throughput sequencing technologies have proven pivotal in elucidating the roles of lncRNAS in human cancers and their interactions with miRNAs, mRNAs and DNA [82]. Apart from being biomarkers, lncRNAs such as HOST2 are now known to play important roles in EMT transition, proliferation and gemcitabine resistance in PDAC patients [83,84]. Colon cancer associated transcript 2 (CCAT2) is a lncRNA that has been associated with tumor growth and chromosomal instability [85]. Recent studies have demonstrated the involvement of CCAT2 in bladder cancer [86], breast cancer [87] and gastric cancer tumorigenesis [88]. In addition, CCAT2 was shown to be upregulated in PDAC tissue and its upregulation was associated with poor patient survival [85].

### 2.5. Public Databases

Databases harboring methylome and epigenome data from resected tumors and biopsy samples have unlocked new avenues for genome-wide epigenetic research. The International Cancer Genome Consortium (ICGC) [89] and The Cancer Genome Atlas (TCGA) [90,91] databases provide access to hundreds of human pancreatic tumor samples for which omics data has been generated. ICGC data utilizing the HumanMethylation450 BeadChip technology has been used to analyze genome-wide methylation patterns in PDAC, employing Spearman’s correlation of differentially methylated CpGs [92,93]. In addition, the ICGC database houses 2800 cancer whole genome data on “somatic mutations, somatic structural variations, copy number alterations, germline variations, RNA expression profiles, gene fusions and phenotypic annotations”, and researchers have conducted pan-cancer analyses on these data [94]. Similarly, TCGA data on global methylation showed differential methylation of markers for pancreatic cancer survival [89]. These databases have made it possible for researchers to study global methylation and gene expression patterns with higher statistical power as a progressively larger number of datasets become available on a common platform.

## 3. Sample Collection Considerations

Tissue biopsies are traditionally used to evaluate epigenetic changes in tumors. However, the inability of tissue biopsies to completely characterize tumors has shifted attention to new diagnostics and minimally invasive techniques, like liquid biopsies. Liquid biopsies involve the analysis of analytes from biological fluids, namely blood [95,96]. The most common analytes investigated in liquid biopsies include circulating tumor cells, cell-free DNA, cell-free RNA and extracellular vesicles, such as exosomes. Circulating tumor cells and tumor DNA not only give insight into the genomic mutations and copy number alterations, but are now regularly used to generate information about the epigenomes [97], transcriptomes [98] and the metabolomes [99] of tumors. The prognostic value of exosomes has been identified, but their application in determining predictive markers and an applicable treatment response remains to be elucidated [100,101]. Recently, liquid biopsies were used to detect tumor-specific methylation changes in cancer patients well before clinical diagnosis of breast [102] and ovarian cancer [103]. 

PDAC tumors are known to be very heterogeneous and include a vast amount of stroma. Therefore, capturing gene expression data on a PDAC tumor will only provide an overview of the transcriptional activity of the tumor and not necessarily reflect what is occurring in the cancer cells. Approaches have been developed to elucidate the cell-to-cell variability in tumors, including PCR amplification of complimentary DNA from a single cells [104,105]. With the advent of more sophisticated technologies, single-cell sequencing has been applied to identify cell types [106], map gene regulatory pathways [107,108] and trace cellular response to stimuli [109]. Recently, single-cell sequencing approaches have been applied for the genomic and transcriptomic profiling of circulating tumor cells, broadening the applications of liquid biopsies. Single-cell sequencing has proven pivotal in establishing intratumoral heterogeneity and gene signatures in esophageal [84] and breast cancers [110], and in differentiating intraductal papillary mucosal neoplasms (IPMNs), a precancerous ductal cyst, from other pancreatic cysts [111]. In addition, the complexity of IPMNs evolution has been shown to vary by early and late driver gene mutations [112]. The applications of single-cell sequencing have not yet had a large impact in the context of analyzing the epigenome of PDAC tumor cells, however, they do suggest the role of stromal extracellular matrix proteins in creating the necessary microenvironment for metastasizing tumor cells [113].

## 4. Epigenome-Wide Studies for PDAC

Studies investigating candidate epigenomic components and their associations with PDAC are increasing exponentially, with many review articles already published [14,16,114]. Since the primary functional role of epigenetic factors is thought to be gene regulation, most studies will focus on the correlation of epigenetic marker levels with gene expression. Below, we focus on summarizing the studies which have taken an epigenome-wide approach to marker identification as a way to make comparisons across studies more consistent. The search and article selection criteria can be found in the methods section of this article.

When genome-wide associations are investigated, numerous statistical tests are performed, therefore, multiple testing adjustment approaches such as the Bonferroni correction [115] or the false discovery rate (FDR) method [116] need to be used to reduce the amount of results that are significant due to chance. The use of setting log2 fold change cutoff values is a technique that some researchers have used to reduce the chance of false negatives, however, the selection of the cutoff is non-standardized. It has been proposed that the use of both p-value and effect size is equally important [117], but the selection of arbitrary cutoffs will result in the exclusion of important alterations. Evidence suggests that cancer is a very heterogeneous disease, with growth and development likely driven by multiple alterations that would be minimal in effect size, rather than a few alterations of large effect size, especially when the effect sizes represent an averaged value for a tissue sample [118].

### 4.1. Methylation

EWAS studies looking at methylation and PDAC are the most common because of the early implementation of technologies. As a consequence, researchers have a better understanding of the functional role that methylation plays in transcription and gene expression compared to other epigenetic factors. The key characteristics of the reviewed methylation EWAS studies are presented in Table 1.

The eight reviewed studies have focused on comparing methylation marker patterns for cancer to either normal or cell line samples. The sample sources were generally from pancreas tissue, but also included blood, cell lines and mouse models. The earlier studies had small sample sizes, limited methylation markers and poor genome coverage. All studies experienced difficulty with the validation of markers, multiple testing and clinical factor adjustments. In the later studies, significance was based not only on the *p*-value but also the beta value, and the methylation level was correlated to gene expression, thereby reducing the likelihood of false-positives and increasing the chances of identifying functionally relevant results. Any methylation markers appearing in multiple studies would be candidates for further study, since issues such platform technology, sample selection and statistical methods would be minimized.

### 4.2. Non-Coding RNA (ncRNA)

NcRNAs, originally considered “transcriptional noise”, are classified into two groups (short (sncRNA) and long (lncRNA)) based on the length of the RNA fragment [119]. These RNAs are able to modify other epigenetic processes by interacting with DNA, other RNA and transcriptional factors. To date, the limited EWAS studies have focused on lncRNA and are presented in Table 2.

The six reviewed studies have focused on comparing lncRNA markers for cancer to either normal or cell line samples. Among these studies, there was a much heavier focus on treatment response and survival as outcomes. The sample sources were generally from pancreas tissue, but also included cell lines and mouse models. All studies have been published since 2017 and have experienced difficulty with the validation of markers, multiple testing, clinical factor adjustments and determining the functional role of identified lncRNA markers. Any lncRNA markers appearing in multiple studies would be candidates for further study, since issues such platform technology, sample selection and statistical methods would be minimized.

### 4.3. Multi-Omics Studies

With the availability of public EWAS datasets with reasonable sample sizes for PDAC, researchers have started to take a more system-wide approach to understanding the complexity and interconnectedness of several types of epigenetic markers. These datasets are an important source for exploratory studies or the validation of potentially important marker sets. The key characteristics of the reviewed studies are presented in Table 3.

The four reviewed studies have focused on comparing at least two marker datasets (e.g., methylation, mRNA, expression, lncRNA) for cancer to either normal or cell line samples. The sample sources were generally from pancreas tissue or cell lines. These types of studies for PDAC started appearing in the literature around 2015 and have benefited immensely from the availability of data through the public databases. Multi-omic studies experience difficulty with the use of non-standard statistical/bioinformatic approaches, multiple tests, clinical factor adjustments and arbitrary filters to reduce dimensionality [120,121,122]. Any marker set appearing significant in these studies has generally appeared in multiple public datasets and in at least two different types of epigenetic marker sets.

## 5. Discussion

To date, EWAS studies have mostly focused on assessing the relationship between PDAC diagnosis or prognosis with methylation or ncRNA markers (both individually and as sets) across the genome. The hypothesized role of epigenetic markers is to regulate gene expression by controlling access to DNA (e.g., presence will block access), and markers are often correlated with gene expression. However, recent evidence suggests that this widely held assumption may not always hold true, as transcription factors have been shown to bind to both methylated and unmethylated DNA [136].

The assessment of epigenetic markers associated with PDAC has been performed for both diagnostic [126,128] and prognostic purposes [122,137]. In general, it has been and will continue to be very challenging to develop potential epigenetic marker sets which provide diagnostic accuracy and reliability for PDAC risk among an asymptomatic population. Focusing on higher-risk subgroups of the population should help, but difficulties will still remain related to the heterogeneity and rarity of PDAC, as well as the effectiveness of existing treatments with earlier diagnoses.

Multi-omic approaches are going to be key to furthering our biological/mechanistic understanding of the development and metastatic behavior of PDAC. Studies have started to identify cell subtypes based on epigenetic signatures in PDAC, and these subtypes have differential survival rates and responses to treatment (reviewed in Reference [93]). Through further research, common heterogeneity PDAC profiles and associated effective combination treatments can be developed to improve overall PDAC patient survival with an individualized approach [135,138,139].

Sample collection and selection are key factors that require transparent discussion of strengths, limitations and study hypotheses whenever researchers are presenting study results. For example, the methylation marker sets that we measured from the cell-free DNA that was collected from a PDAC patient will tell us different information than the methylation marker set which we would measure from the primary tumor or in circulating exosomes in the same patient [16,140]. In terms of the samples collected from patients, availability will be limited to those who have a diagnosis (meaning generally a later stage diagnosis), thereby limiting the length of time to impact survival and the ability to understand early, fundamentally important alteration/mutations and develop effective early detection biomarkers. Mouse models (e.g., patient-derived xerographs), with their own limitation, are a proposed approach of testing treatment effectiveness and understanding key early changes in the pancreas leading to PDAC [141]. The importance of these differences and the utility of the less complete and less invasive processes have yet to be determined but hold much promise.

Candidate gene/pathway studies in the laboratory using multiple tissues/cells will continue to provide further biological and mechanistic evidence of statistical associations observed at the genome-wide level. These type of laboratory studies will also provide insight into biological pathways and can use knockout/knockdown models to clarify the functional relationships observed through statistical associations and computational modeling [142].

Genome-wide based technologies and techniques are creating new datasets at a fast pace. As scientific researchers, we need to be conscious and always highlight the key limitations of the sample collection and lab-based methods, along with the results we are presenting. Each sample type and technology provides us with a snapshot view of the biological system and disease state. Therefore, attempting to further understand the biological processes and mechanisms of a complex and heterogeneous disease like PDAC will require us to take a more systematic approach through the integration of multi-omic datasets and laboratory results.

## 6. Materials and Methods

A search was conducted using the database PubMed in order to identify all published studies with a combination of the following keywords in the abstract or title: “Pancreatic adenocarcinoma”, “pancreatic cancer”, “methylation”, “RNA”, “epigenetic”, “multi-omics”, “epigenomic”, “epigenomes-wide”, “genome-wide”. Abstracts were then screened to determine which articles were EWAS studies for full review and inclusion in Table 1 or Table 2. The first step resulted in 295 articles. The articles where then excluded for one of the following reasons: (1) Not related to PDAC; (2) not an EWAS study; or (3) not in English. There were 17 studies that underwent full review for inclusion in Table 1, Table 2 and Table 3.

## Figures and Tables

**Table 1:** Epigenome-wide association studies (EWAS) identifying methylation markers associated with pancreatic ductal adenocarcinoma (PDAC).

Source	Tissue Type	Techniques	Sample Size	Comparisons	Strengths	Weakness
[123]	Pancreas tissue	88K Agilent promotor array and 244K island array—methylated CpG island amplification (MCA)	10 pancreatic cancer cell lines; normal human pancreatic ductal epithelium (HPDE) and human pancreatic Nestin-expressing cells (HPNE)	Cancer vs. normal	● Study conducted in cells lines and patient tissue	● Early implementation of technology
			57 pancreatic cancer samples and 34 normal pancreas samples		● Investigated using several approaches	● Limited number of probes
[124]	Leukocytes	Illumina GoldenGate methylation Beadchip—1505 CpG sites	Phase 1: 132 never-smoker cases and 60 never-smoker controls	Cancer vs. normal	● Validation	● Limited number of probes
			Phase 2: 240 cases and 240 matched controls (half never smokers)		● Adjustment for some confounders	● Promotor region CpGs only
[54]	Cell lines and pancreas tissue samples	244K ChIP-on-Chip microarray—27800 CpG island array	9 pairs of pancreatic cancer versus normal pancreatic epithelial tissues	Cancer vs. normal	● Looked at number different cell lines and tissue samples	● CpG islands only
			3 matched pairs of pancreatic cancer versus lymphoid tissue from same individual		● No validation within this study	● Looked at methylation difference as individual samples rather than average of population
[125]	Pancreas tissue samples	Methyl capture sequencing method—(methylCap-Seq)	10 pancreatic cancer tissues and 10 adjacent non-tumor tissues	Cancer vs. normal	● Explored potential functional result of CpG methylation	● Used *p*-value < 0.05
					● 728/3911 differently methylated genes identified that were also reported in at least one of 3 different studies	● Early implementation of technology
[92]	Pancreas tissue samples	Infinium 450k methylation array (Illumina)	167 untreated PDACs and 29 adjacent normal pancreata	Cancer vs. normal	● Larger sample size	● No discussion of the significance of dissimilar pathway analysis results using two different methods
			121 PDAC and 8 nontumor	Survival	● Looked at methylation across potential confounding factors	● Survival analysis methods not described
					● 850/3522 genes previously reported to have differential methylation	
					● Determined significance based on p-value and beta value	
[126]	Pancreas tissue samples	HumanMethylation450k Beadchip (Illumina)	Secondary analysis of public TCGA data - 184 tumors and 10 normals	Cancer vs. normal	● Looked at methylation and expression, as well as mutation loads and copy number variations of key oncogenes or suppressor genes	● Had to attempt to adjust for batch effects using PCA
					● Promoter region methylation highly negatively correlated with gene expression	● Used median beta value for genes with multiple methylation markers with no justification
					● Non-promoter region methylation highly positively correlated with gene expression	● Stated gender bias was ignored by excluding X and Y chromosomes
					● Determined significance based on p-value and beta value	● Used only beta value for significance
					● Highlighted methylation of genes coding for other epigenetic markers	
[127]	PDX – pancreas tissues - stem cells	HumanMethylation450k Beadchip (Illumina)	Not given	Cancer stems cells vs. non-cancer stem cells	● Looked at stem cells from PDAC-185, liver met (PDAC-A6L) and single cell-derived tumor	● Unknown systematic effects of DNMT1 treatment
					● Function of stems cells reduced by inhibiting DNMT1	● Unknown sample size used
					● Cancer stem cells show hypermethylation in intergenic regions	
[128]	PDX – pancreas tissue	Chip-seq	24 xenograft samples - tumor	Survival	● Looked at chromatin states, DNA methylation, Gene expression, and Transcription factors	● limited to later stage samples
		RNA-seq				
		MethEpic				

**Table 2:** EWAS studies identifying ncRNA markers associated with PDAC.

Source	Tissue Type	Techniques	Sample Size	Comparisons	Strengths	Weakness
[129]	Pancreas tissue	Affymetrix Human Genome U133 Plus 2.0	Secondary analysis: 117 tumor samples and 73 normal pancreas samples	Cases vs. control	● Two markers validated in independent cohort	● Set significance at log2 fold change > 1 and *p*-value < 0.05
		Agilent-014850 Whole Human Genome Microarray	Independent set: 145 tumor and 46 normal samples		● Multiple platforms used	
		IlluminaHiSeq	165 samples from TCGA	Survival		
[130]	Pancreas tissue	RNA-seq	29 pancreatic ductal adenocarcinoma xenogragts	Drug targets	● Used public databases and patient-based samples	● Most functional impacts unknown
		miRNA-seq	3 public databases			
[128]	PDX—Pancrease tissues	Chip-seq	24 xenograft samples - tumor	Survival	● Looked at chromatin states, DNA methylation, gene expression, and transcription factors	● Limited to later stage samples
		RNA-seq			●	
		MethEpic			●	
[131]	Pancreas tissue—cell line and mouse	RNA-seq	4 E1A;HRasV12;Neat1+/+ and 4 E1A;HRasV12;Neat1−/−	Gene expression	● Used multiple mouse and human cells	● Literature has contradictory role for Neat1
		Chip-seq			● Demonstrated important functional roles for Neat1	● Previous evidence of Neat1 role in tumorigenesis is unclear
					Implication related to p53	
[132]	Pancreas tissue	RNA-seq	Mouse	Gene expression	● Associated Arid1a with MyC	● Previous evidence of ARID1A role in tumorigenesis is unclear
	Cell lines	Chip-seq	Pancreatic ductal epithelial cells		● Different roles given pancreatic cancer cell type	● Mutational profiles of IPMN currently unknown
[133]	Cell lines		11 cell line from patient-derived xenografts	Gene expression	● GATA6 regulated epithelial-mesenchymal transition	● Proposed new functional role of an EMT regulator
	Pancreas tissue samples		25 tumor samples	Survival	● Patients with low GATA6 have worse survival and worse treatment response	● Prior evidence for functional roles in other cancers
				Treatment response	● Used samples from randomized clinical trial	● GATA6 suspected oncogene, but patients with low expression have worse outcomes
					● Support role of GATA6 in tumor differentiation	● No cause-effect relationship with 5-FU treatment response

**Table 3:** Multi-omic EWAS studies identifying marker networks in PDAC.

Source	Tissue Type	Techniques	Sample Size	Comparisons	Strengths	Weakness
[121]	Pancreas tissue	5617 miRNA—Affymetrix GeneChip miRNA 3.0	104 PDAC and 17 benign pancreas tissue	Cancer vs. benign	● Candidate markers annotated using gene ontology analysis	● New approach - unvalidated
		33,297 mRNA—HuGene 1.0 ST	Validation in GEO and TCGA databases	Cancer vs. normal	● Selection of genes based on predictive measures and adjusted p-values	● Weights are dataset dependent, however, limited markers to validation in at least 2 datasets
[134]	PDAC tumor tissue and cell lines	exome—llumina HiSeq 2000)	3 different cell lines and 6 primary pancreatic cancer tumors	Primary tumor vs cell lines	● Combined exome data and transcriptome data	● Variant analysis and interpretation
		transcriptome—RNA-seq (Illumina HiSeq 2000)			● Variant filtering in pipeline removes most false positives	● Biopsy samples generally included normal tissue
					● Made analysis pipeline available for others to try and establish standard and reproducibility	● Exome only on cell lines
[122]	Pancreas tissue	multiple—Table 1 in reference		Cancer vs. normal	● Used FDR to determine significance	● Datasets with no class-based clustering were excluded
				Survival	● Focused meta-analysis on functional markers	● Several arbitrary filters applied - currently no standardized data combining techniques
					● Visualization of significant results	● Clinical factors not taken into account in survival plots
					● Large sample size - meta analysis	● Hard to identify causal changes
[135]	Cell lines	Agilent Human Whole-genome expression microarray	3 BxPC-3 and 3 BxPC-3ER	Treatment response	● Investigated specific expression changes associated with erlotinib resistance using BXPC cell line	● Understanding metabolite changes is limited
					● Identified potential metabolic pathways and associated genes to target to counter resistance	● Expression and phosphorylation of RTKs not consistent with previous reports
